# Modern Therapy of Septic Puerperal and Surgical Infections

**Published:** 1903-06

**Authors:** Roswell Park

**Affiliations:** Buffalo, N. Y., Professor of Surgery, University of Buffalo


### ABSTRACT.

### Modern Therapy of Septic Puerperal and Surgical Infections.

BY ROSWELL PARK, M. D., LL.D., Buffalo, N. Y.,
Professor of Surgery, University of Buffalo.

[*Alpha Omega Delta Bulletin, March, 1903.*]

THE author says that the most efficient measures for the treatment of surgical infections are the various silver preparations, for whose introduction into surgical and obstetrical work we are indebted to Credé, of Dresden. We have been for decades looking in vain for an effective antiseptic which is devoid of marked toxic or irritating properties. Allotropic silver (collargolum) seems to offer us the nearest approach thereto. Between this silver preparation, which is so bland, and the silver salts, like nitrate of silver, there are the lactate and citrate of silver, also introduced by Credé, of which reasonably strong solutions can be used upon quite sensitive surfaces without producing much if any disturbance.

Let us first take the aqueous solution of soluble metallic silver (collargolum), which in the strength of 1 to 500 in distilled water makes a somewhat cloudy solution. In this strength it may be used by intravenous injection in cases of severe general or puerperal sepsis, rapidly spreading gangrene, acute articular rheumatism or other serious infections. In fact, solutions as strong as 1 to 100 may be employed, it being desirable to introduce 6 cg. (.9 grain) to 10 cg. (1½ grains) at least. If there be difficulty in injecting it into a vein it may even be given beneath the skin. Unpleasant effects will not be noticed, neither will any immediate relief follow, but the solution thus introduced coming into contact with the blood, which in these cases is swarming with germs, will promptly begin its bactericidal work, whose effects should be manifested after two or three hours by a fall of temperature and amelioration of septic symptoms. Silver used in this way has been of great service in cases of carbuncle and even of acute anthrax. Moreover, its administration may be repeated as often as may seem necessary.

When metallic silver is made into a suitable ointment (unguentum Credé) which, by the way, much resembles mercurial ointment, and is then applied to the skin, there is a rapid absorption of the silver itself with its dissemination into the blood stream and results like those just mentioned. It is simply a somewhat slower method of introducing it into the system. For many years I held and taught that the combination of resorcin, ichthyol and mercurial ointment, which I believe I introduced into surgical practice, was the most effective remedy for the treatment of erysipelas and all similar septic infections. Today I have found but one combination which I think superior for this purpose, and that is the silver ointment, unguentum Credé. I believe that its properties are more marked than those of the ointment which I so long used. No matter what part of the body be anointed absorption takes place readily and promptly, consequently any convenient surface may be medicated in this way. Cleanse the skin thoroughly, smear the ointment freely over the surface, cover the area with oiled silk, and put over this, if comforting to the patient, a warm application to promote absorption. If the surface be not tender, the ointment may be rubbed in. In cases of puerperal sepsis it may be applied over the abdomen or to the inside of the thighs. In erysipelas it should be applied to the affected part.

Lastly, I would speak of the use of lactate and citrate of silver, not only for such purposes as the preparation of catgut, silk, gauze, etc., but in solutions of from 1 to 300 to 1 to 500 for the irrigation of septic cavities, and for such purposes as washing out the peritoneal cavity in cases of tuberculous peritonitis, for which I have repeatedly used it and always with benefit.

In my own experience, in several instances, a flushing of that cavity with a 1 to 500 solution has been of the greatest apparent benefit and has never occasioned any regret. Infected bladders, uterine cavities and vaginas may be advantageously, freely and frequently washed out with similar solutions. When using them one may have the feeling that he is using solutions of greater efficacy and of far less toxicity than any of the mercurial preparations would afford. Therein lies the beauty of these preparations, that in anything like equal strength they are more effective and much less toxic than the mercurial salts.

I often state in my clinic that the good old-fashioned nitrate of silver is not used nearly so much as it should be and prove the strength of my conviction by its general use in 1 to 10 per cent. solutions in pus cavities. Not only is a full germicidal effect obtained but also that stimulation to healthy granulation which the nitrate is well known to afford. All in all, if I could have but one source for antiseptic solutions and applications, I would rather look toward the preparations of silver than in any other direction.

---

Never allow an ice-bag to remain for many hours upon the skin without careful inspection. Very severe frost-bites may occur in patients, particularly when their circulation is impaired, through the agency of such intense cold applied continuously, as is so often done in pelvic and appendicular inflammations and in strangulated hernia.—*International Journal of Surgery.*

